# Antimicrobial Composite Films Based on Alginate–Chitosan with Honey, Propolis, Royal Jelly and Green-Synthesized Silver Nanoparticles

**DOI:** 10.3390/ijms26146809

**Published:** 2025-07-16

**Authors:** Corina Dana Dumitru, Cornelia-Ioana Ilie, Ionela Andreea Neacsu, Ludmila Motelica, Ovidiu Cristian Oprea, Alexandra Ripszky, Silviu Mirel Pițuru, Bianca Voicu Bălașea, Florica Marinescu, Ecaterina Andronescu

**Affiliations:** 1 Department of Science and Engineering of Oxide Materials and Nanomaterials, Faculty of Chemical Engineering and Biotechnology, National University of Science and Technology Politehnica Bucharest, 011061 Bucharest, Romania; corina.dumitru95@yahoo.com (C.D.D.);; 2 National Research Center for Micro and Nanomaterials, National University of Science and Technology Politehnica Bucharest, 060042 Bucharest, Romania; ludmila.motelica@upb.ro; 3 Academy of Romanian Scientists, 3 Ilfov Street, 050044 Bucharest, Romania; 4 Research Center for Advanced Materials, Products and Processes, National University of Science and Technology Politehnica Bucharest, 313 Splaiul Independentei, 060042 Bucharest, Romania; 5 Department of Inorganic Chemistry, Physical Chemistry and Electrochemistry, Faculty of Chemical Engineering and Biotechnologies, National University of Science and Technology Politehnica Bucharest, 060042 Bucharest, Romania; 6 Interdisciplinary Center for Dental Research and Development, “Carol Davila” University of Medicine and Pharmacy, 6 Traian Vuia Str., 020956 Bucharest, Romania; 7 Department of Biochemistry, Faculty of Dental Medicine, “Carol Davila” University of Medicine and Pharmacy, 17-23 Plevnei Street, 020021 Bucharest, Romania; 8 Department of Organization, Professional Legislation and Management of the Dental Office, Faculty of Dental Medicine, “Carol Davila” University of Medicine and Pharmacy, 17-23 Plevnei Street, 020021 Bucharest, Romania; 9 The Research Institute, University of Bucharest, 050663 Bucharest, Romania; florica.marinescu@bio.unibuc.ro; 10 Department of Botany and Microbiology, Faculty of Biology, University of Bucharest, 060101 Bucharest, Romania

**Keywords:** green synthesis, Ag nanoparticles, honey, propolis, royal jelly, biomaterials, wound healing, antibacterial, antifungal

## Abstract

Honey, propolis or royal jelly are considered natural remedies with therapeutic properties since antiquity. Many papers explore the development of antimicrobial biomaterials based on individual bee products, but there is a lack of studies on their synergistic effects. Combining honey, propolis and royal jelly with silver nanoparticles in a biopolymer matrix offers a synergistic strategy to combat antibiotic-resistant bacterial infections. This approach supports progress in wound healing, soft tissue engineering and other domains where elimination of the microorganisms is needed like food packaging. In this study we have obtained antimicrobial films based on bee products and silver nanoparticles (AgNPs) incorporated in an alginate–chitosan blend. The novel biomaterials were analyzed by UV-Vis, fluorescence and FTIR spectroscopy or microscopy, SEM and thermal analysis. Antibacterial tests were conducted against both Gram-positive and Gram-negative bacteria, while the antifungal properties were tested against *Candida albicans*. The diameters for growth inhibition zones were up to 10 mm for bacterial strains and 8 mm for the fungal strain. Additionally, cytotoxicity assays were performed to evaluate the biocompatibility of the materials, the results indicating that the combination of honey, propolis, royal jelly and AgNPs does not produce synergistic toxicity.

## 1. Introduction

Wound healing is a complex and dynamic process that restores tissue integrity and function, occurring in three distinct phases. The initial phase, named hemostasis and inflammation, involves the formation of a blood clot and the release of growth factors from platelets. It also includes increased vascular permeability, along with the migration of immune cells (neutrophils, monocytes, macrophages and lymphocytes) to the wound site, where they release cytokines to prevent infection. During the proliferative phase, granulation tissue forms, composed of blood vessels, fibroblasts and inflammatory cells [1]. This phase also includes neovascularization, re-epithelialization and wound contraction, which contribute to tissue regeneration. In the final phase, known as maturation and remodeling, the granulation tissue is gradually replaced by mature collagen, leading to the completion of the healing process [2].

Blocking topical bacterial infections, removing wound exudates (by absorption) and keeping an optimal moisture level at the wound surface are mandatory properties for an ideal wound dressing material [3]. At the same time, the wound dressing should be biocompatible, non-toxic and non-allergenic and should promote tissue regeneration with the end objective of supporting the wound healing, while allowing for painless removal [4]. The dressing should also offer antibacterial properties, thermal insulation and a soft texture to enhance comfort and effectiveness [5].

Polymeric hydrogels are beneficial for wound care as they maintain a moist environment and absorb excess fluids [6]. Among them, polysaccharide-based hydrogels stand out for being non-toxic, biodegradable and biocompatible, effectively promoting re-epithelialization and speeding up wound closure [7]. Alginate, a natural polysaccharide derived from algae, is commonly used in hydrogel wound dressings due to its biocompatibility, affordability and versatility in applications such as tissue engineering and drug delivery [8]. However, alginate alone has limited biological activity, so it is often combined with other bioactive substances like honey, propolis, herbal extracts, royal jelly or silver to enhance its healing effects [9,10].

Chitosan, a widely used polysaccharide, is especially effective due to its ability to stop bleeding, stimulate fibroblast growth, support blood vessel formation, ensure proper collagen distribution and boost natural hyaluronic acid production at the wound site [11]. Additionally, incorporating antibacterial agents into these hydrogels enhances healing by protecting wounds from bacterial infections [12].

Wound infections are often linked to bacterial colonization and biofilm formation, particularly in non-healing cases. As a result, effective wound healing typically requires antimicrobial treatment [13]. Due to rising infection rates and growing antibiotic resistance, alternative therapies are being explored. Silver-based biomaterials, especially those containing silver nanoparticles (AgNPs), are emerging as promising candidates for wound dressings in both research and clinical settings [14].

There is a pressing need for new antimicrobial agents that are cost-effective, non-toxic to normal cells and minimize bacterial resistance. Green-synthesized agents should also exhibit high efficacy against antibiotic-resistant bacteria [15]. Silver nanoparticles represent a classic antimicrobial solution in the biomedical field due to their broad-spectrum activity against pathogenic bacteria and minimal toxicity to mammalian cells. Additionally, nanosilver-based materials serve as effective prophylactic and therapeutic agents to prevent wound colonization by microorganisms [16].

Bee products such as honey, royal jelly and propolis have recently emerged as promising natural agents in nanotechnology, particularly for the synthesis of silver nanoparticles [17].

The honeybees have a remarkable ability to convert nectar into honey, a substance historically used for both nutrition and medicine since prehistoric times. Honey is rich in flavonoids and polyphenols, which contribute to its strong antioxidant and anti-inflammatory effects. It also exhibits antimicrobial and wound-healing properties, largely due to components such as hydrogen peroxide, methylglyoxal, polyphenols and bee defensin-1 [18]. For centuries, honey has been applied to accelerate the healing of ulcers, bedsores, burns and other skin infections [19].

In addition to honey, the bees produce other valuable substances like royal jelly [20] and propolis [21]. These bee products have long been recognized for their nutritional benefits and their role in supporting health and well-being [22].

Propolis is a highly promising natural material for advanced wound dressings, thanks to its broad biological activities, including antibacterial, antifungal, antiviral, anti-inflammatory and anticancer properties [23]. It can mimic the skin’s extracellular matrix and support the wound healing process. When combined with natural polymers like chitosan, which offer high biocompatibility and promote cell growth, propolis-based materials become more adaptable and effective for biomedical applications [24].

Royal jelly, a nutrient-rich secretion from honeybees, contains proteins, lipids, flavonoids and phenolic compounds that contribute to immune support, digestion and glucose regulation, making it a beneficial additive for wound care [25].

**Figure 1:**
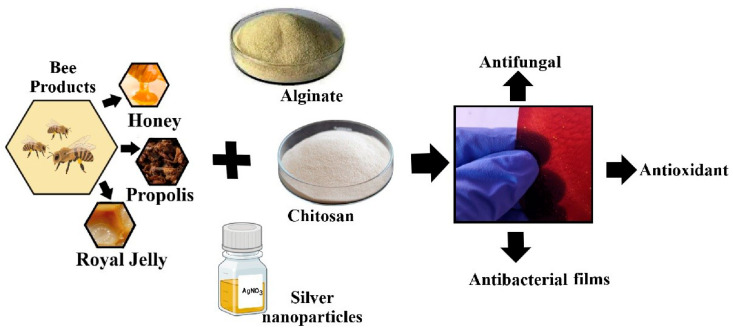
The schematic idea of the present research.

This paper centers on honey and other bee-derived products—propolis and royal jelly with AgNPs and particularly their combination—an area that has seen limited exploration in the existing literature [26]. To our knowledge, this is the first comprehensive study on honey, propolis, royal jelly and AgNP mixtures within any polymeric matrix, not only alginate or chitosan. Given the lack of studies on their synergistic effects, the aim was to offer new insights into enhancing the therapeutic potential of bee-product-based formulations. This is achieved by incorporating AgNPs and examining their impact on the overall effectiveness of these natural compounds (Figure 1).

## 2. Results and Discussion

### 2.1. Scanning Electron Microscopy (SEM)

**Figure 2:**
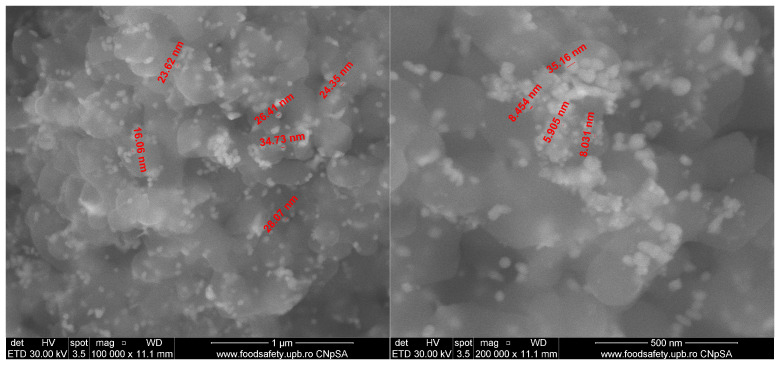
SEM micrographs (at magnifications 100,000× on the left and 200,000× on the right) of AgNPs obtained in propolis tincture (red are dimensions of the measured AgNPs).

The synthesis of AgNPs was followed by SEM analysis of a dried sample collected from the solution (Figure 2). The micrograph is revealing the presence of quasi-spherical nanometric silver particles, with a size between 5 and 35 nm on the surface of the dry propolis. The AgNPs are well dispersed, with only minor agglomeration tendency.

**Figure 3:**
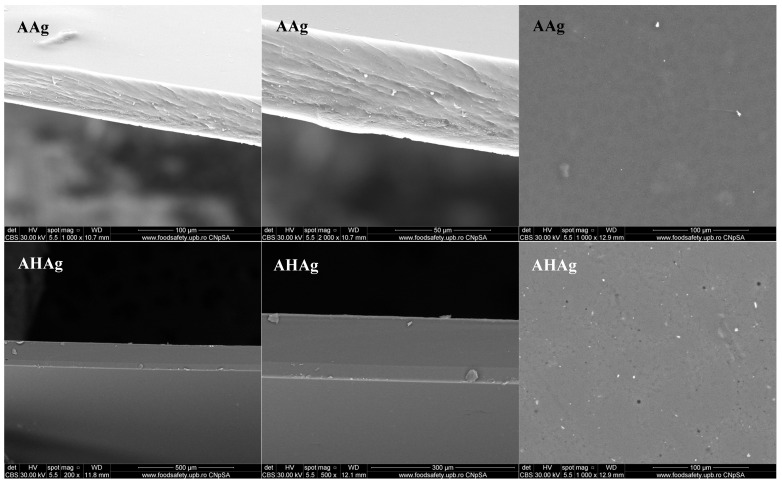

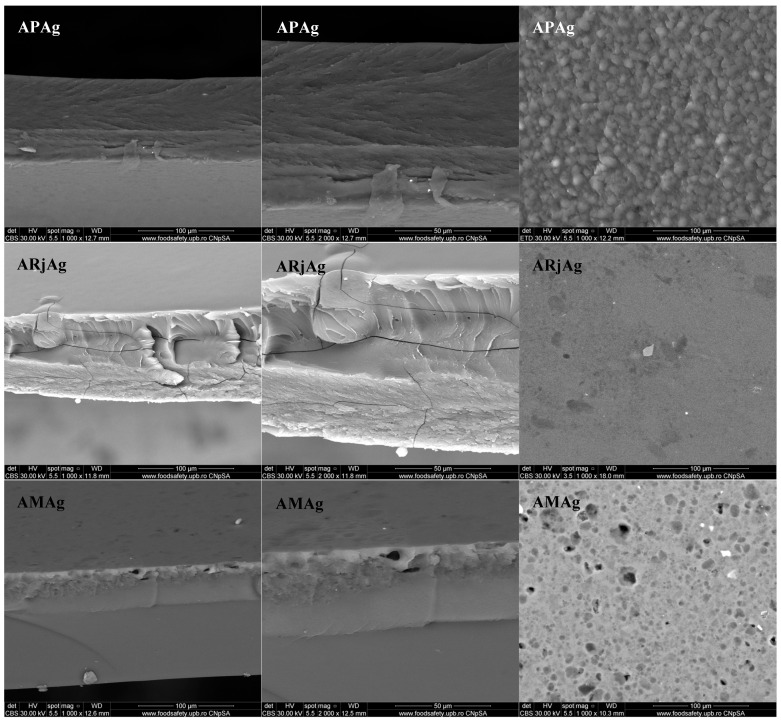
SEM micrographs for A; AHAg; APAg; ARjAg and AMAg samples. In each row the left and middle micrographs are for cross-sections, and the right micrograph represents the top view on the composite films.

SEM analysis of the sodium alginate–chitosan composites show clear morphological variations between the different formulations (Figure 3). For each sample we have performed a SEM analysis on the surface and in the cross-section (after cryofracture in liquid N_2_). Due to silver nanoparticle (Ag) incorporation into the honey-based matrix (AHAg), the surface displays scattered bright spots, indicating the presence of nanoparticles, while maintaining an overall uniform distribution. The sample combining propolis with Ag (APAg) presents a more irregular texture, with nanoparticles seemingly interacting with the bioactive compounds, potentially leading to enhanced structural complexity. The royal-jelly-infused formulation (ARjAg) has also a layered appearance, with a fibrous appearance, and a rather smooth surface, with minor heterogeneities, indicating a good dispersion of AgNPs, contributing to increased porosity and textural variation [27].

The presence of Ag in this multi-component system (AMAg) further accentuates these variations, leading to a porous and structurally complex surface, where nanoparticle dispersion is evident.

Finally, the incorporation of silver into the pure alginate–chitosan matrix (AAg) results in a relatively uniform structure, with well-distributed nanoparticles subtly altering the surface properties. The silver particles appear as bright spots, maintaining the integrity of the polymer network while likely enhancing its antimicrobial properties.

Overall, SEM analysis revealed that the inclusion of bee products and AgNPs notably affected the morphology of the sodium alginate–chitosan composites, resulting in differences in surface roughness, porosity and structural features among the various formulations.

### 2.2. Fourier Transformed Infrared (FTIR) Spectrometry

**Figure 4:**
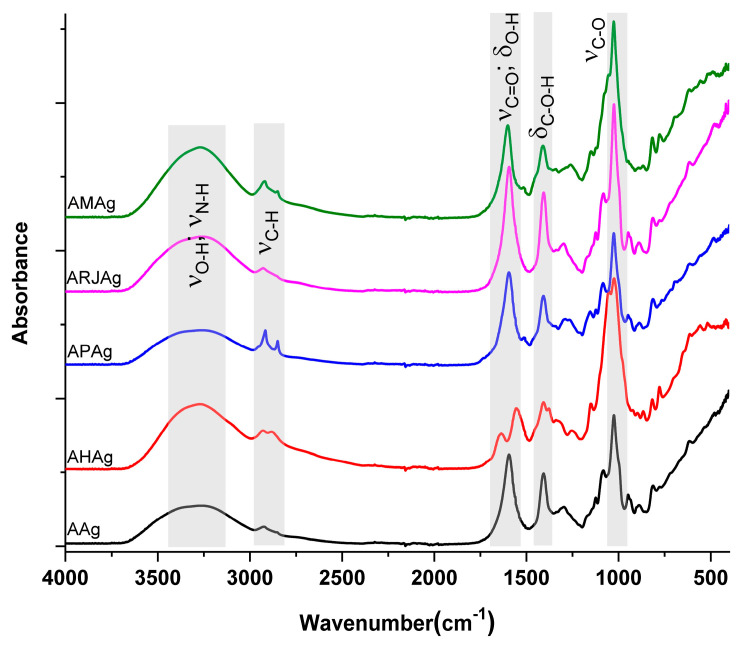
FTIR spectra for AAg; AHAg; APAg; ARjAg; AMAg samples.

The FTIR analysis of the sample with honey revealed significant absorption signals at 3277, 2926, 1624 and 1024 cm^−1^, corresponding to key functional group vibrations. The broad band with maximum at 3277 cm^−1^ is attributed to N-H and O-H stretching from chitosan, alginate and bee products [28]. The spectral region between 3000 and 2800 cm^−1^ indicates vibrational modes characteristic of a C-H bond in carbohydrates, amino acids and carboxylic acids, which are essential components of honey [29]. The asymmetric and symmetric C-H stretching vibrations are visible at 2926 and 2860 cm^−1^ and originates from all organic compounds from samples [30]. At the same time the absorption peak from 1624 cm^−1^ is associated with O–H deformation combined with C=O stretching (amide I band) from chitosan and proteins. Additionally, in the AHAg sample, the amide II band assigned to the deformation of –NH_2_ moiety can be observed as a separate peak at 1552 cm^−1^. The band from 1412 cm^−1^ is assigned to the in-plane bending vibration of the hydroxyl group O-H, while the peak at 1024 cm^−1^ is assigned to stretching vibration for C-OH bond and C-O-C vibration in polysaccharides [31]. The peak from 885 cm^−1^ is specific for the β configuration of the anomeric carbon in glucosamine unit of the chitosan [32]. These spectral features (Figure 4) provide valuable insights into the protein composition and structural characteristics of the sample, reinforcing the significance of FTIR analysis in identifying key biomolecular components [33].

FTIR analysis reveals that propolis is rich in hydrocarbons, phenolic compounds, terpenes and aromatic hydrocarbons, with lower levels of proteins and carbohydrates. In contrast, royal jelly contains high amounts of carbohydrates and proteins but lower levels of lipids and terpenoid compounds [34].

**Figure 5:**
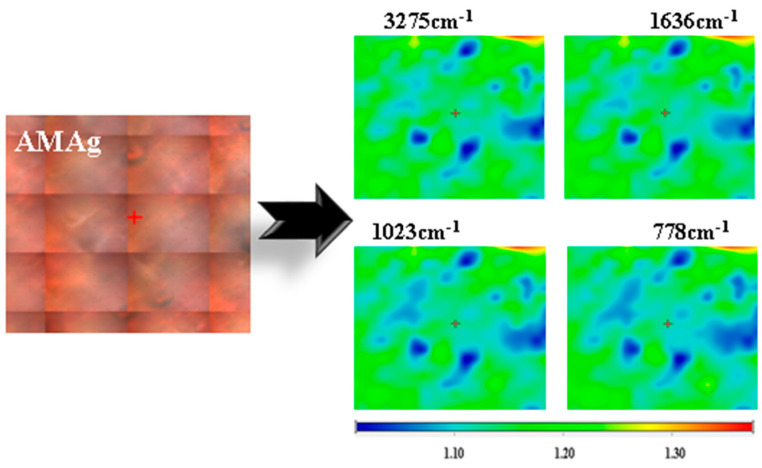
FTIR maps at 3275 cm^−1^, 1636 cm^−1^, 1023 cm^−1^, 778 cm^−1^ for the AMAg sample. Red areas indicate the highest absorbance, while blue areas correspond to the lowest absorbance.

The FTIR microscopy maps for samples without AgNPs are presented in Figure S1 and for samples with AgNPs are presented in Figure S2. The samples AP and APAg exhibit the most evident differences between maps, indicating the existence of local agglomerations for the waxy/resinous components into the hydrophilic polymeric matrix. In Figure 5 are presented the FTIR 2D maps recorded for wavenumbers (ν_O-H_ 3275 cm^−1^, ν_C=O_ 1636 cm^−1^, ν_C-O-C_ 1023 cm^−1^ and 778 cm^−1^), the specific bonds being present in both polysaccharides, alginate and chitosan, but also in honey and other organic compounds. The FTIR 2D maps are similar at all wavenumbers indicating that the polymer blends are homogenous, the films presenting only minor thickness variation.

### 2.3. UV-Vis and Fluorescence Spectral Analyses

The UV-Vis spectrum for the green-synthetized AgNPs is presented in Figure S3. The AgNPs, like all metallic nanoparticles, present a characteristic peak, generated by the surface plasmon resonance phenomena. The wavelength of the maximum gives information about the shape and size of the nanoparticles, while the broadness of the band indicates the size distribution [35]. The sample exhibit a broad band, with the peak at 433 nm, indicating that the preferred shape is spherical for the AgNPs [36]. The absorption band is quite large, hinting that the nanoparticles have a broad size distribution, confirming the data from the SEM analysis [37,38].

**Figure 6:**
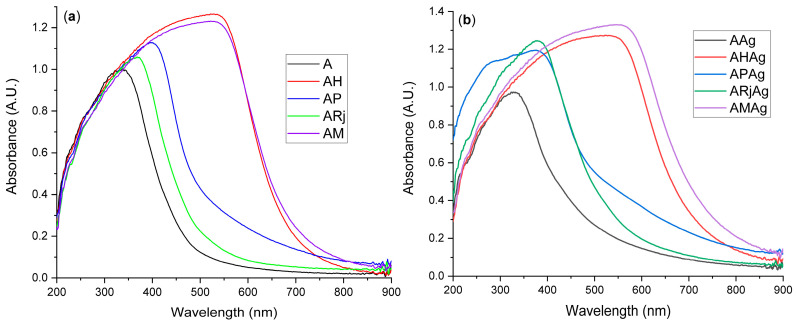
The UV-Vis spectra for the samples without AgNPs (**a**) and with AgNPs (**b**).

The UV-Vis spectra are presented in Figure 6 for both kind of samples, with or without AgNPs. A complete comparison between samples with and without AgNPs is presented in Figure S4.

The addition of propolis to the alginate–chitosan film (AP sample) leads to an increase in the absorbance and bathochromic shift of the absorption maximum, from 336 nm to 397 nm. However, the addition of AgNPs further increases the absorbance and shifts back the absorbance maximum to 376 nm, with a shoulder at 276 nm, as a clear indication of the interactions between propolis and AgNPs. In the case of the royal jelly sample (ARj) a similar increase in absorbance and a bathochromic shift are observed (from 336 nm to 369 nm) but with a smaller magnitude than those from AP sample. As Rj has a different chemical composition, with proteins and lipids, the introduction of AgNPs leads to a different interaction, and as a consequence the ARjAg absorbance increases strongly and shifts further to 376 nm.

The introduction of honey leads to a broader absorbance band on the whole visible spectrum. Consequently, the AM spectrum resembles more with that from the AH sample, the contribution of propolis and royal jelly being practically masked. The sample AMAg has a broader and higher intensity than AHAg most probably due to the strong influence of royal jelly and propolis and AgNPs interactions, as seen also in ARjAg and APAg samples.

The introduction of AgNPs in the AHAg sample leads to a shift of the absorption maximum from 530 nm to 535 nm and a minor increase in intensity (Figure S4). Some influence on the intensity of the absorption bands can be assigned to scattering generated by the different surface roughness and particle content, and therefore by UV-Vis spectrometry we cannot assess the interactions between AgNPs and honey in AHAg sample. At the same time, in the case of AMAg the same shifting tendency can be observed, from 527 nm (AM) to 553 nm (AMAg), coupled with a significant increase in the absorption intensity. These modifications can be assigned to the interactions between AgNPs and components of propolis and royal jelly, as seen in APAg and APARj spectra (Figure S4).

Both chitosan and alginate, as polysaccharides, exhibit a strong fluorescence emission when in solid state [28,39,40]. This feature is characteristic to polysaccharides in solid phase due to a mechanism reported firstly in 2001 [41]. While no traditional fluorophore is present in alginate or chitosan, the polymeric chains have many electron-rich heteroatoms, like oxygen or nitrogen, which in solid phase come in close vicinity and generate electron clouds that are overlapped and shared, thus generating novel clustered electron-rich chromophores with lowered energy gaps and extended effective conjugation lengths [42,43]. Such a mechanism is called aggregation-induced emission (AIE) and is believed to be the cause of the fluorescence for non-aromatic polymers like alginate, starch, chitosan or cellulose and even phosphorescence [44]. Chitosan and alginate fluorescence was previously reported in the literature [45,46]. In solid form the vibrational dissipations are blocked, thus giving enhanced fluorescent emission [44].

**Figure 7:**
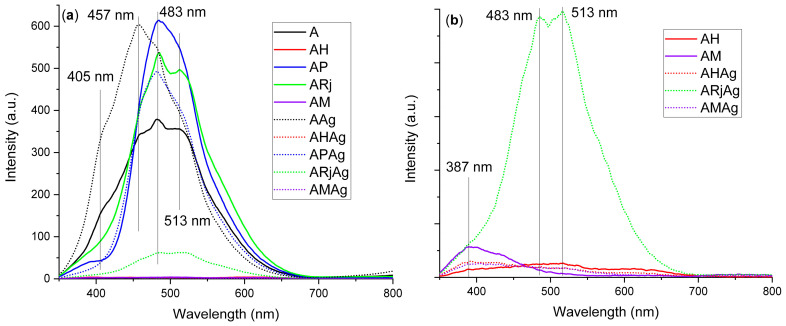
The fluorescence spectra for all samples (**a**) and a zoom-in view for the low florescence samples ARjAg, AH, AHAg, AM and AMAg (**b**).

For the sample AAg, a hypsochromic shift of the strong emission band, from 483 nm to 457 nm, is observed, confirming the interactions among polymeric components and AgNPs. Introduction of propolis or royal jelly has increased the intensity of the fluorescence emission from 483 nm. On the other hand, the introduction of honey has practically totally quenched the fluorescent emission of the polymer matrix (AH and AM), a feature that is also observed for the samples with AgNPs (AHAg and AMAg). A similar behavior is reported in [47], where authors found that mannose addition is quenching chitosan fluorescence. Additionally, in [48] it is reported that the alginate fluorescent emission is quenched by the glucose addition. Therefore, we can conclude that the presence of simple sugar molecules (glucose and fructose from honey) is the main factor inhibiting the fluorescence of the AHAg and AMAg samples (Figure 7).

The fluorescence emission is also diminished for the sample ARjAg as an indicator of the interaction between royal jelly components (proteins and lipids) with the AgNPs, confirming the observations from UV-Vis spectrometry. Lastly, despite strong emission of the AAg (457 nm) and AP (483 nm) samples, the APAg sample exhibits a lower intensity of the 483 nm emission band and no shoulder for the 457 nm emission, indicating strong interactions between AgNPs and propolis’ components.

### 2.4. Thermal Analysis

**Figure 8:**
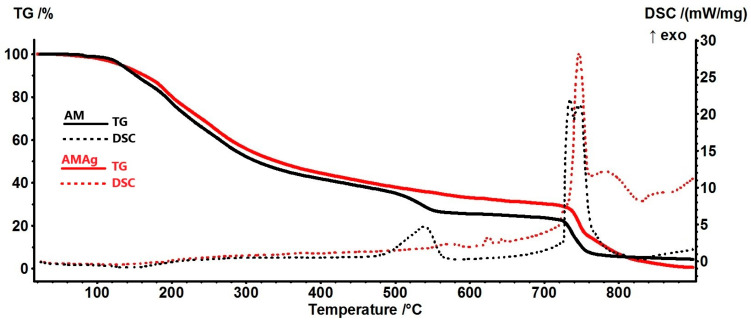
Thermal analysis for AM (black curves) and AMAg (red curves) samples: the TG curves are represented as solid lines, and the DSC curves are represented as dotted lines.

The thermal analysis was performed on blended samples with and without AgNPs, the results being presented in Figure S5. A typical comparison is given in Figure 8 for AM and AMAg samples.

Both samples are losing residual solvent molecules up to 115 °C [28], the recorded mass loss being ~2–3%. The FTIR of the evolved gases for the AMAg sample indicates that the carbon dioxide evolving (from oxidation reactions) is starting later than for the AM sample (198 °C vs. 112 °C, respectively) [49]. Between 115 and 460 °C the main degradation event takes place, in a single mass loss step, representing 56.45%. In this temperature interval, polysaccharides are dehydrated, proteins are degraded and the polymers’ backbone is fragmented. At the same time, the more labile moieties and smaller resulted fragments are oxidized. Therefore, this step can be seen as an overlap of decomposition and oxidation processes that leads to no clear thermal effect on the DSC curve.

**Figure 9:**
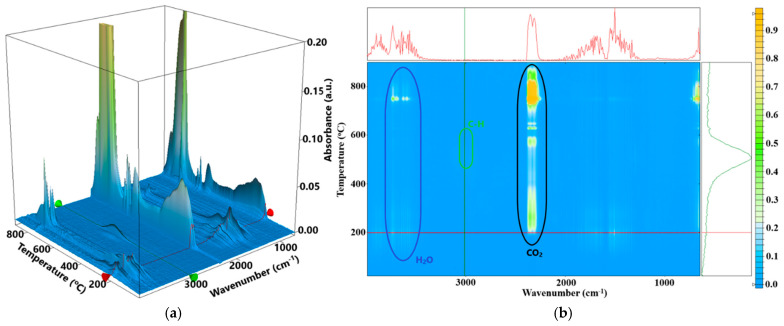
The FTIR 3D diagram for the gases evolved from thermal analysis of the AMAg sample (**a**) and the 2D projections in the temperature/wavenumber plane (**b**). On top of the 2D projection is the FTIR spectrum at the temperature of 198 °C, corresponding to the red line in the 3D diagram; at the right side of the 2D projection is the evolving trace for the wavenumber 3010 cm^−1^ assigned to the C_sp2_-H vibration from unsaturated hydrocarbon fragments, corresponding to the green line from the 3D diagram.

In Figure 9 are presented the FTIR 3D diagrams for the evolved gases and the 2D projection in the plane temperature/wavenumber. These diagrams indicate that between 115 and 460 °C the evolved gases contain H_2_O and CO_2_ but also minor quantities of CO.

Comparing the intensities for the characteristic vibrations of C=O and –OH we conclude that the most likely reactions are the dehydration of polysaccharides, followed by the partial oxidation of branched moieties and smaller fragments [40]. The characteristic C-H stretching bands can be observed in the FTIR spectrum only at temperatures higher than 460 °C. This is a clear indication that up to this temperature the fragmentation rate is lower than the oxidation rate and that a burst in the fragmentation process is taking place around 460 °C.

The recorded mass loss between 460 and 720 °C is 11.01%, and the FTIR 3D diagram (Figure 9) indicates less CO_2_ evolved when comparing with the results reported in [49], and therefore the oxidative processes are occurring with smaller intensity. This is confirmed by the weak exothermic peaks from the DSC curve, with maxima at 571.8 and 625.2 °C.

The complete oxidation of the residual carbonaceous mass occurs in the interval 730–750 °C, associated with a very strong exothermic effect on the DSC curve at 746.6 °C [39]. The characteristic CO_2_ vibration bands at ~2350 cm^−1^ are presented in the FTIR of evolved gases together with H_2_O stretching and bending bands, confirming an oxidation process after 720 °C.

The principal numeric data are summarized in Table 1. By comparing the values for the temperatures at which 1%, 5% or 10% of the sample mass has been lost (T1%, T5% and T10%) a clear trend can be observed between samples with AgNPs and without them. The addition of AgNPs leads in general to the increase in Tx% suggesting a stabilizing effect induced by the interactions between AgNPs and the polymeric matrix or other organic compounds.

### 2.5. Antimicrobial Activity

The biological activity of the alginate–chitosan films was first assessed for their antimicrobial properties. Antimicrobial effectiveness was evaluated qualitatively by measuring the growth inhibition zone diameters (GIZDs) around the samples, with results reported as mean values ± standard deviation.

**Figure 10:**
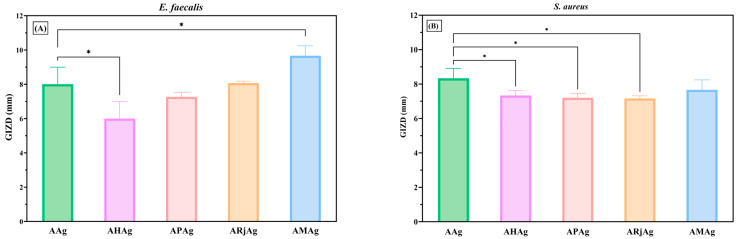
Antibacterial profiles of alginate–chitosan films against Gram-positive bacteria: (**A**) *E. faecalis* ATCC 29212 and (**B**) *S. aureus* ATCC 25923. The differences between AAg (control) and samples with bee products were statistically analyzed in one-way ANOVA and Dunnett’s multiple comparisons tests. The data are considered statistically significant (* *p* ≤ 0.046).

The biomaterials based on bee products and AgNPs demonstrated moderate antibacterial activity, effectively inhibiting the growth of both Gram-positive bacterial strains (Figure 10A,B). However, the higher GIZD values of samples enriched with bee products than the control (AAg) were observed only in the case of *E. faecalis*.

Moreover, the film containing a mixture of honey, propolis, and royal jelly and incorporated silver nanoparticles (AMAg) exhibited the highest antimicrobial efficacy, as reflected by the largest inhibition zone diameter, indicating a possible synergistic effect when all three bee-derived substances are combined. The chitosan–alginate film containing only silver nanoparticles (AAg) also demonstrated strong antibacterial activity, confirming the well-documented antimicrobial properties of silver [50].

Moderate inhibition was observed in the samples containing either royal jelly (ARjAg) or propolis (APAg) in combination with silver nanoparticles, both showing similar GIZD values. In contrast, the film incorporating honey and AgNPs (AHAg) exhibited the lowest antibacterial activity among the tested formulations, although it still showed a statistically significant inhibitory effect compared to the control. These findings suggest that combining natural bee products with AgNPs can enhance inhibitory effects against *E. faecalis*, with the mixture-based formulation (AMAg) emerging as a promising candidate for antibacterial wound dressings.

The inhibition zones (GIZD) for all tested formulations against *S.aureus* show relatively similar diameters, ranging between approximately 6 and 7 mm. The AAg formulation shows the highest inhibition zone, suggesting that AgNPs incorporated in alginate–chitosan film exert slightly greater antibacterial effects against *S. aureus* than the combinations with bee products. The other formulations (AHAg, APAg, ARjAg and AMAg) exhibit low, nearly equivalent antibacterial activity, indicating that the addition of honey, propolis, royal jelly, or their combination does not enhance the antibacterial effect against *S. aureus* in this context. Overall, while all tested films demonstrate antibacterial efficacy, no statistically significant differences appear among them, and AgNPs alone remain the primary contributor to bacterial inhibition in the case of *S. aureus* [51,52].

**Figure 11:**
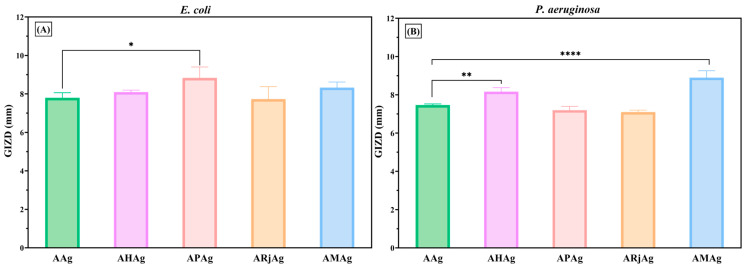
Antibacterial profiles of alginate–chitosan films against Gram-negative bacteria: (**A**) *E. coli* ATCC 25922 and (**B**) *P. aeruginosa* ATCC 27853. The differences between AAg (control) and samples with bee products were statistically analyzed in one-way ANOVA and Dunnett’s multiple comparisons tests. The data are considered statistically significant (* *p* < 0.044; ** *p* ≤ 0.008 and **** *p* ≤ 0.0001).

Figure 11A illustrates the antibacterial activity of chitosan–alginate films enriched with AgNPs and various bee products against *E. coli*, a Gram-negative strain. Among the tested formulations, the APAg sample exhibited the highest inhibitory effect, showing a statistically significant difference compared to AAg. The second most inhibitory effect was that of the AMAg sample, which seems to be slightly higher than the control (AAg). Although the other formulations AHAg and ARjAg also demonstrated moderate zones of inhibition, they did not significantly outperform the control. These results indicate that propolis, when combined with AgNPs, enhances antimicrobial efficacy against *E. coli* more effectively than honey or royal jelly. These results are in concordance with a recent study, which reported that propolis determined a higher sensitivity of strains and are followed by royal jelly and honey [53].

The graph from Figure 11B presents the antibacterial activity of chitosan–alginate films incorporating various bee products and AgNPs against *P. aeruginosa*. Among the tested formulations, the composite containing the mixture of all three bee products and silver nanoparticles (AMAg) showed the largest inhibition zone, indicating the strongest antibacterial effect. This suggests a possible synergistic interaction between the combined bee products and AgNPs in enhancing antimicrobial efficacy. Likewise, the significant inhibitory effects of AMAg is linked to a higher concentration of bee products (Table 2), which are the biologically active compounds.

The honey-based film (AHAg) also exhibited a significantly higher antibacterial effect compared to the base film with silver alone (AAg), emphasizing honey’s notable antimicrobial contribution. Meanwhile, films containing individual bee products such as propolis (APAg) and royal jelly (ARjAg) demonstrated moderate but consistent inhibition, though to a lesser degree than AMAg. Statistical significance is marked, showing meaningful differences particularly between AAg and AHAg (**) and between AAg and AMAg (****), highlighting the improved efficacy when combining natural bioactive compounds with AgNPs in combating *P. aeruginosa*.

**Figure 12:**
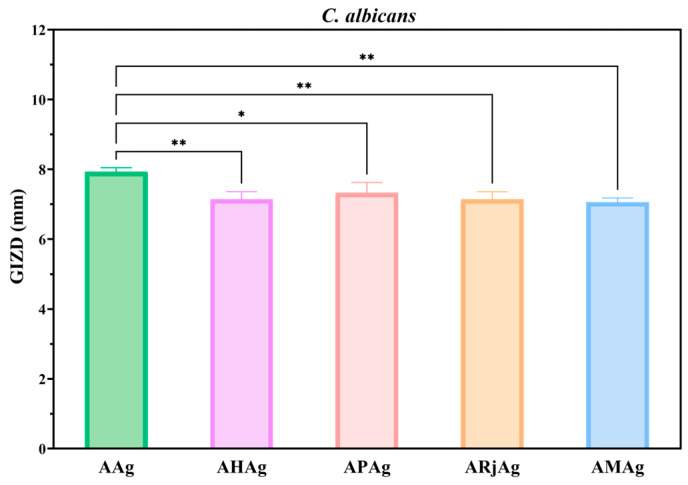
Antimicrobial profiles of alginate–chitosan films against *Candida albicans*. The differences between AAg (control) and samples with bee products were statistically analyzed in one-way ANOVA and Dunnett’s multiple comparisons tests. The data are considered statistically significant (* *p* < 0.0188 and ** *p* ≤ 0.0080).

In the case of *Candida albicans*, all film formulations demonstrated relatively similar antifungal effects, with inhibition zones ranging from approximately 7.5 to 8.5 mm (Figure 12). The control film (AAg) showed the highest GIZD, suggesting that silver plays a key role in antifungal activity. However, the addition of individual bee products—honey (AHAg), propolis (APAg) and royal jelly (ARjAg)—as well as their combination (AMAg), did not significantly enhance or diminish the antifungal efficacy compared to AAg.

The lower GIZD values among the formulations implies that, while bee products are known for their bioactive properties, their contribution to antifungal activity against *C. albicans* in this composite matrix may be limited or comparable to the control. This result highlights the dominant role of AgNPs in combating fungal strains within these biopolymer-based films.

Lower antimicrobial activity of some of the samples can also be explained by the strong interactions with the AgNPs as indicated by the UV-Vis and fluorescence spectra. These interactions are partially blocking the antimicrobial expression of AgNPs, which can lead to a slower release and a prolonged activity.

Since these results are from qualitative screening, future studies will involve quantitative assessment of the antimicrobial activity, especially the anti-adhesion ability of these materials. Analyses will also be conducted to examine the release behavior of bioactive compounds and their impact on the growth of microbial cultures in liquid media. Furthermore, the assays will be performed to highlight possible synergistic or antagonistic effects between the compounds used for developed materials.

### 2.6. Biocompatibility and Cytotoxicity Assays

#### 2.6.1. MTT Assay

The MTT assay assesses cell viability by detecting metabolic activity (higher readings reflect greater cell survival and proliferation, whereas lower values indicate potential cytotoxic effects).

The AAg sample, consisting of chitosan–alginate film with AgNPs, exhibited high cell viability, nearly equivalent to the control (~100%). This result supports previous studies demonstrating the good biocompatibility of low-concentration AgNPs embedded in biopolymeric matrices, particularly when stabilized by natural polymers such as chitosan and alginate [54,55].

In contrast, the incorporation of bee products altered the cytotoxicity profile. The AHAg sample showed a pronounced decrease in cell viability to approximately 60%, while the APAg and ARjAg samples (incorporating propolis and royal jelly, respectively) also demonstrated moderately reduced viability levels around 60–65%. These reductions suggest that certain bioactive components in these substances, such as phenolic acids, flavonoids and organic acids, may induce metabolic stress or interact unfavorably with cellular membrane [56,57].

Notably, the AMAg formulation, which combined all three bee products with AgNPs, resulted in the lowest observed cell viability, approximately 50%. This may reflect a cumulative or synergistic cytotoxic interaction among the diverse bioactive compounds present in the bee-derived components when used together. It also indicates the need for careful balancing of the concentration to avoid adverse effects on host cells, particularly fibroblasts critical to wound healing

**Figure 13:**
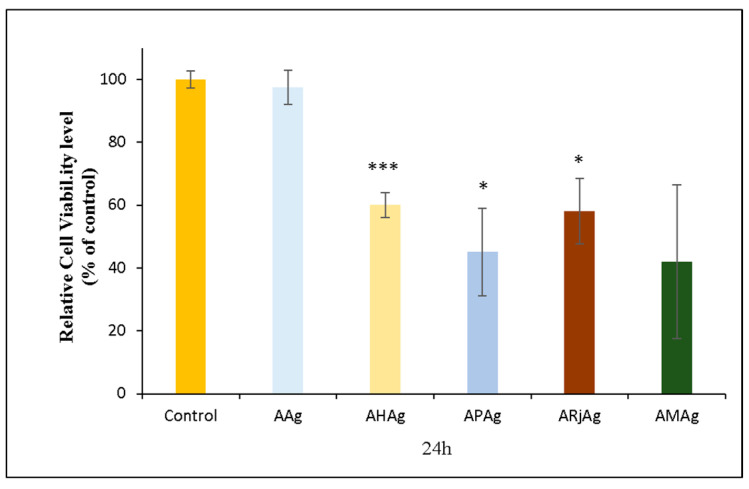
The relative cell viability of human gingival fibroblasts (HFIB-G cell line) measured by MTT assay after 24 h of incubation with medium previously incubated for 24 h with the samples based on bee products: AAg, AHAg, APAg, ARjAg and AMAg. Results are expressed as means ± standard deviation (SD) (n = 3) and represented relative to the control (untreated cells). * *p* < 0.05 and *** *p* < 0.001 compared to control.

Figure 13 illustrates the percentage of viable cells relative to the control for various chitosan–sodium alginate films enriched with bee products and AgNPs.

In summary, while AgNPs alone appear cytocompatible, the addition of specific bee products, particularly when combined, can significantly reduce cell viability. These findings emphasize the importance of carefully optimizing the concentration and combination of natural ingredients in bioengineered films to ensure a favorable balance between antimicrobial effectiveness and cellular compatibility in wound healing applications.

#### 2.6.2. NO Assay

The nitric oxide (NO) assay results offer important information on the immunomodulatory and possible antimicrobial effects of chitosan–sodium alginate films enriched with bioactive bee products and AgNPs. Since nitric oxide is a key mediator in immune regulation, inflammation and tissue repair, the measured NO levels help assess the films’ potential impact on immune responses and wound healing processes [58].

All experimental groups exhibited a statistically significant increase in nitric oxide (NO) production compared to the control, underscoring the potential immunostimulatory properties of these biofunctionalized biomaterials. Among them, the APAg sample, containing propolis and AgNPs, demonstrated the highest NO levels, exceeding 300% relative to the control. This finding aligns with the well-documented immunomodulatory and antimicrobial potential of propolis, which is rich in phenolic compounds and flavonoids known to stimulate immune cell responses [59,60].

**Figure 14:**
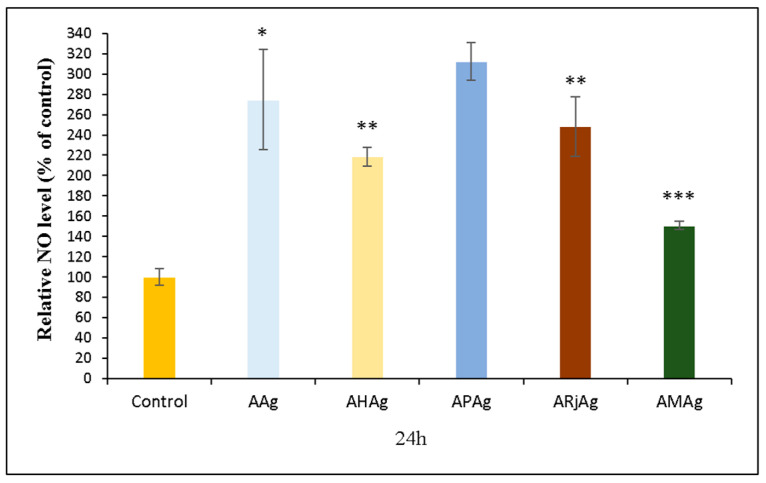
Relative NO level of human gingival fibroblasts (HFIB-G cell line) cells measured by NO assay after 24 h of incubation with medium previously incubated for 24 h with the samples based on bee products: AAg, AHAg, APAg, ARjAg and AMAg. Results are expressed as means ± standard deviation (SD) (n = 3) and represented relative to the control (untreated cells). * *p* < 0.05, ** *p* < 0.01 and *** *p* < 0.001 compared to the control.

The AAg formulation also significantly elevated NO levels (approximately 260%), suggesting that AgNPs alone can stimulate NO production—likely due to mild cellular stress or activation of defense mechanisms (Figure 14). Similarly, AHAg, ARjAg and AMAg films also triggered elevated NO responses, though to varying extents.

Interestingly, AMAg, the film containing the full mixture of bee products, showed a comparatively lower NO induction (~160%), despite its composite nature. This attenuation may result from a complex interplay between the bioactive compounds in honey, propolis and royal jelly. Their combined presence could have modulated each other’s individual effects, leading to a more balanced, less pronounced NO release. Alternatively, it may suggest that the formulation is better tolerated by cells, leading to reduced immune stimulation [61].

These findings are significant because nitric oxide plays a crucial role in immune defense, inflammation and tissue repair, making NO levels a valuable indicator of the immunological impact of biomaterials. Elevated NO production suggests enhanced immune activation, which can contribute to antimicrobial defense and wound healing. However, excessively high NO levels can also indicate cellular stress or inflammation, so the balance observed in AMAg might be desirable for applications where controlled immune modulation is needed [62].

#### 2.6.3. LDH Assay

**Figure 15:**
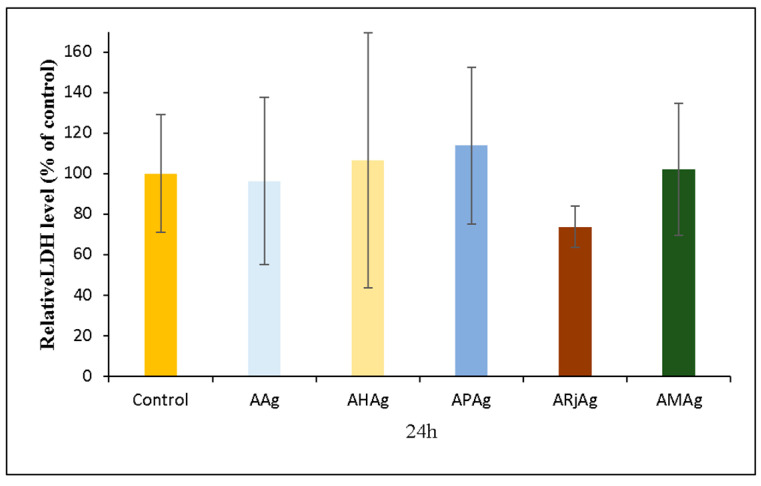
Relative LDH level of human gingival fibroblasts (HFIB-G cell line) cells measured by LDH assay after 24 h of incubation with medium previously incubated for 24 h with the samples based on bee products: AAg, AHAg, APAg, ARjAg and AMAg. Results are expressed as means ± standard deviation (SD) (n = 6) and represented relative to the control (untreated cells).

The cytotoxic potential of the chitosan-sodium-alginate-based films incorporating AgNPs and bee products was assessed using the lactate dehydrogenase (LDH) release assay in human gingival fibroblasts (HFIB-G cell line). The assay quantitatively measures membrane damage by detecting the release of LDH enzymes from compromised cells. The results, presented in Figure 15, indicate that none of the tested formulations induced significant LDH release compared to the untreated control.

These findings suggest that all samples maintain cellular membrane integrity after 24 h of exposure, indicating low cytotoxicity and good biocompatibility. Notably, the ARjAg samples exhibited the lowest relative LDH release, suggesting a potential membrane-stabilizing effect of royal jelly, which is consistent with its known antioxidant and cytoprotective properties [63].

Similarly, the AHAg and APAg films demonstrated LDH levels comparable to the control group, supporting the hypothesis that flavonoid-rich bee-derived compounds do not exert detrimental effects on membrane integrity [64,65]. The AMAg formulation, comprising a mixture of all three bee products, also maintained LDH levels within the biocompatibility threshold, indicating that the combination does not produce synergistic toxicity. This aligns with recent evidence supporting the cytocompatibility of natural-product-enriched films for applications in wound care and oral regeneration [66].

The samples based on honey, propolis and royal jelly showed reduced metabolic activity according to the MTT assay, but the LDH results indicated that cell membranes were still intact. MTT (3-(4,5-dimethylthiazol-2-yl)-2,5-diphenyl-2H-tetrazolium bromide) is a mono-tetrazolium salt that enters viable cells due to its positive charge and lipophilic structure. In metabolically active cells, MTT is reduced to an insoluble violet compound called formazan. This redox reaction forms the basis of the MTT assay and is widely used to assess cellular metabolic activity [67]. Because the MTT test mainly reflects mitochondrial enzyme activity—not cell death—this suggests that the cells may be experiencing aerobic metabolic intensity fluctuations rather than real toxicity. One possible explanation could be the caloric excess in the form of carbohydrates from honey, propolis and royal jelly, whether tested individually or in combination, which may have led to a downregulation of the aerobic glucose oxidation pathway (glycolysis—Krebs cycle—mitochondrial respiratory chain). This pathway includes enzymes (such as succinate dehydrogenase) responsible for the conversion of MTT reagent into formazan crystals. This mechanism has been outlined by Ghasemi M. et al. in [67], suggesting that the presence of serum in the culture medium may influence the cells’ nutritional status, intracellular signaling, metabolic activity and pathways, as well as oxidative stress levels, cell cycle progression, viability and proliferation.

Overall, these results corroborate findings from recent studies highlighting the safety of biopolymer matrices embedded with AgNPs at controlled concentrations [68].

## 3. Materials and Methods

### 3.1. Materials

Key materials including chitosan with low molecular weight, silver nitrate with purity > 99%, sodium alginate and ethanol (90%) were sourced from Sigma-Aldrich (Darmstadt, Germany). Natural products such as black locust (pseudoacacia) honey, royal jelly and propolis were obtained from a personal apiary. The propolis tincture was macerated for 48 h after adding 100 g of propolis in 1000 mL ethanol (90% *v*/*v*).

The AgNPs were obtained using a green method with AgNO_3_ as a precursor and propolis tincture as a reducing agent.

Antimicrobial evaluations were conducted using Nutrient Broth No. 2 (NB), Agar (microbiological grade) and Sabouraud Glucose Agar (Sab), both obtained from Sigma-Aldrich (Darmstadt, Germany). All microbial strains used in this study were provided by the Microorganism Collection of the Department of Microbiology, Faculty of Biology, University of Bucharest.

### 3.2. Synthesis of Biomaterials Based on Bee Products

A mixture of natural bioactive compounds was prepared using black locust honey (35 g), propolis tincture (20 mL) and royal jelly (5 g), then homogenized in a water bath for 20 min, and stored in sealed, dark conditions.

To develop sodium alginate–chitosan films, 1 g of sodium alginate and 0.1 g of glycerol were dissolved in 50 mL of distilled water, to which either honey, propolis tincture, royal jelly or the prepared mixture was added. These solutions were cast into Petri dishes and vacuum-dried at 35 °C for 24 h.

Subsequently, a chitosan solution (2 g chitosan in 50 mL of 1% acetic acid) was prepared. A 10 mL layer of this solution was added to each dried sodium alginate film, followed by a second vacuum drying step at 35 °C for another 24 h. The result was a series of bilayer films combining natural bioactive compounds with biopolymer matrices.

For enhancing the biological properties of the films, silver (Ag) nanoparticles were incorporated into them by using a green synthesis method with the propolis tincture as a reducing and stabilizing agent [17]. Preparation of Ag NPs colloid included the following: A solution of silver nitrate (AgNO_3_) was prepared by dissolving 0.0168 g of AgNO_3_ in 99 mL of distilled water under continuous stirring. To adjust the pH of the solution, 5 drops of sodium hydroxide (NaOH) solution were added gradually, ensuring the final pH reached 9.6. After achieving the desired pH, 1 mL of propolis tincture was added to the solution; the mixture was stirred continuously to facilitate the reduction in silver ions (Ag^+^) to silver nanoparticles (AgNPs). A color change in the solution was monitored as an indicator of nanoparticle formation.

Finally, the samples with the following composition were obtained: AAg or the control, AHAg (3.5 g of honey), APAg (2 mL of propolis tincture), ARjAg (0.5 g of royal jelly) and AMAg (5 mL of mixture) (Table 2). The results for the samples without AgNPs (A, AH, AP, ARj and AM) were previously reported in [49].

### 3.3. Characterization of Biomaterials Based on Bee Products

A scanning electron microscope (SEM), QUANTA INSPECT F50 (FEI Company, Eindhoven, Netherlands) was used to investigate the surface morphology and microstructure of the films, as well as the formation of AgNPs.

A Nicolet iS50 FTIR spectrophotometer (Thermo Fisher Scientific Inc., Madison, WI, USA) was used to obtain the FTIR spectra in the range 400 cm^−1^ to 4000 cm^−1^. All spectra were recorded in ATR mode, with a resolution of 4 cm^−1^, as a 32 scans average.

A Nicolet iN10 MX FTIR microscope (Nicolet, Waltham, MA, USA), with a liquid-N_2_-cooled MCT detector, was used to record the FTIR 2D maps in the range 650–4000 cm^−1^.

A simultaneous thermal analysis system Netzsch STA 449 C (NETZSCH Gerätebau GmbH, Selb, Germany) was used to investigate the thermal behavior of the samples in the temperature interval 20–900 °C. The analyses were performed in a dynamic air atmosphere (50 mL/min), with a heating speed of 10 °C/min. A complex thermal analysis system is composed from a TG-DSC device, STA 449C F3 (NETZSCH Gerätebau GmbH, Selb, Germany), coupled with an FTIR Tensor 27 from Bruker (Bruker Co., Ettlingen, Germany) which was used for evolved gases analysis.

A JASCO V560 spectrophotometer (JASCO Inc., Easton, PA, USA) was used to measure the UV-Vis spectra. The device was equipped with a 60 mm integrating sphere (ISV-469) and a film holder for the samples. The spectra were recorded with a speed of 200 nm min^−1^, in the domain 200–900 nm.

A Perkin Elmer (Waltham, MA, USA) LS55 spectrometer was used to measure the photoluminescence spectrum (PL). A Xe lamp was used as a UV light source at ambient temperature, the fluorescence being measured in the range 350–800 nm. The spectra were recorded with a scan speed of 200 nm min^−1^, with excitation and emission slits of 10 nm and a 350 nm cut-off filter. An excitation wavelength of 320 nm was used.

### 3.4. Antimicrobial Assessments

The antimicrobial activity of the samples was qualitatively tested against *Enterococcus faecalis*, *Staphylococcus aureus*, *Escherichia coli*, *Pseudomonas aeruginosa* and *Candida albicans*. To prevent contamination, all film samples (0.5 × 0.5 cm) were sterilized using UV light for 30 min on each side. Sterility was confirmed by incubating the samples in Nutrient Broth at 37 °C for 24 h; the absence of turbidity indicated no microbial growth.

An adapted spot diffusion method, based on the Clinical Laboratory Standards Institute [69] guidelines, was used to assess antimicrobial effects [70]. Standardized microbial suspensions were prepared from 24 h cultures, and inoculated Petri dishes were treated with the test films. After 24 h of incubation at 37 °C, the diameters of growth inhibition zones (GIZD) were measured using ImageJ software (version 1.8.0, National Institutes of Health, Madison, WI, USA).

### 3.5. Biocompatibility Assays

#### 3.5.1. Cell Culture

Human gingival fibroblast cells (HFIB-G), from Provitro (Berlin, Germany), were cultured in 75 cm^2^ flasks using modified Dulbecco’s Modified Eagle Medium (DMEM) supplemented with 10% fetal bovine serum and 1% antibiotic-antifungal solution to promote cell growth and prevent contamination. The medium was refreshed every three days, and the cells were incubated at 37 °C in a humidified atmosphere with 5% CO_2_ to maintain optimal physiological conditions.

#### 3.5.2. Indirect Cytotoxicity Tests

Bee-product-based samples were sterilized with 70% ethanol for 30 min and dried under sterile conditions. Each sample measured 1 mm in height and 7 mm in diameter. HFIB-G were seeded in 96-well plates at a density of 2 × 10^4^ cells per well and allowed to adhere overnight. The next day, the culture medium was replaced with 200 μL of medium previously incubated with the samples for 24 h. Cells were then incubated for another 24 h at 37 °C with 5% CO_2_. A control group was maintained in standard DMEM without sample exposure. After incubation, MTT, lactate dehydrogenase (LDH) and nitric oxide (NO) assays were conducted to evaluate cell viability, cytotoxicity and NO production.

Cell viability was assessed using the MTT assay (Biotium, Fremont, CA, USA, Cat. No. 30006). In this procedure, 10 μL of MTT reagent was added to 100 μL of culture medium per well and incubated for 4 h. After incubation, 200 μL of isopropanol was added to dissolve the formed formazan crystals. Absorbance was measured at 570 nm and 630 nm using the FLUOstar^®^ Omega Multi-Mode Microplate Reader (BMG LABTECH, Ortenberg, Germany).

The LDH Cytotoxicity Assay was used to evaluate cell membrane damage and cytotoxic effects, following the protocol provided with the LDH Cytotoxicity Assay Kit (MAK529, Sigma-Aldrich). In each well, 50 μL of culture medium was combined with 80 μL of the reaction mixture and incubated for 10 min at room temperature in the dark. Absorbance was measured at 500 nm using the FLUOstar^®^ Omega Multi-Mode Microplate Reader (BMG LABTECH, Germany) with Omega software version: 5.70 R2.

Nitric oxide production was measured using the Griess method with the Nitric Oxide Assay Kit, Colorimetric (MAK454, Sigma-Aldrich), following the manufacturer’s instructions. The working reagent was prepared in the recommended ratio (100 μL Reagent A, 4 μL Reagent B, 100 μL Reagent C). Samples were mixed with the reagent in a 1:2 ratio and incubated at 60 °C for 10 min. Absorbance was then read at 540 nm using the FLUOstar^®^ Omega Multu-Mode Microplate reader (BMG LABTECH, Germany).

### 3.6. Statistical Analysis

Antimicrobial data were statistically analyzed using GraphPad Prism 10.4 (GraphPad Software, San Diego, CA, USA). Results are presented as mean ± standard deviation (SD). Differences among alginate–chitosan film groups were evaluated using one-way analysis of variance (one-way ANOVA), followed by Tukey’s or Dunnett’s multiple comparison tests. A *p*-value of less than 0.05 was considered statistically significant.

Experimental data were compared to control values and graphically represented using the arithmetic mean of each test type. Biocompatibility assay results were statistically analyzed in Microsoft Excel, using standard deviation and t-test functions to evaluate variability and determine significance.

## 4. Conclusions

In this study, silver nanoparticles synthesized via a green, propolis-mediated method were obtained and were further incorporated in novel biomaterials developed by combining chitosan and alginate with bee-derived products (honey, propolis and royal jelly). Structural and morphological analyses, including SEM and spectral techniques, confirmed that both the natural additives and silver nanoparticles significantly modified the films’ surface architecture and chemical composition, while maintaining thermal stability up to 100 °C.

The AMAg formulation (with the mixture of bee products and silver nanoparticles) showed the highest inhibition against *E. faecalis*, while AAg (silver nanoparticles alone) was most effective against *S. aureus*. These results suggest that the combination of bee products and silver nanoparticles enhances antimicrobial effects more noticeably against certain Gram-positive strains, particularly *E. faecalis*. APAg and AMAg samples were also effective against Gram-negative bacteria showing similar inhibitory effects for *E. coli* and *P. aeruginosa*.

The cytotoxicity assessment combining MTT, LDH and NO assays demonstrated that while chitosan–alginate films with silver nanoparticles (AAg) exhibit favorable biocompatibility, the incorporation of bee-derived products—particularly in combination (AMAg)—can enhance cellular stress and reduce fibroblast viability. These findings highlight the importance of optimizing the composition and concentration of natural additives to achieve a balance between antimicrobial efficacy and cytocompatibility, which is critical for the safe and effective development of advanced wound healing biomaterials.

## Figures and Tables

**Table 1:** Principal data from the thermal analysis of samples with and without AgNPs.

Sample	T1%	T5%	T10%	Mass Loss RT–115 °C	Mass Loss 115–460 °C	Mass Loss 460–720 °C
A	50.1 °C	94.6 °C	156.7 °C	6.94%	53.18%	21.65%
AAg	45.8 °C	95.0 °C	163.9 °C	6.73%	52.00%	27.09%
AH	60.7 °C	100.9 °C	134.6 °C	6.93%	55.50%	23.41%
AHAg	92.6 °C	134.3 °C	162.4 °C	2.68%	56.07%	24.22%
AP	48.1 °C	87.8 °C	142.5 °C	7.81%	53.22%	23.81%
APAg	57.3 °C	98.8 °C	158.1 °C	6.83%	53.08%	26.28%
ARj	46.1 °C	87.6 °C	144.1 °C	7.67%	52.89%	21.27%
ARjAg	51.4 °C	100.2 °C	159.7 °C	6.36%	54.00%	22.77%
AM	95.3 °C	132.4 °C	152.7 °C	2.03%	59.92%	15.25%
AMAg	80.4 °C	132.1 °C	163.9 °C	3.09%	56.45%	11.01%

**Table 2:** Samples composition and labels.

Sample	Alginate	Honey	Propolis	Royal Jelly	AgNP (mL)	Chitosan	Glycerol
AAg	1 g				2 mL	0.4 g	0.1 g
AHAg	1 g	3.5 g			2 mL	0.4 g	0.1 g
APAg	1 g		2 mL		2 mL	0.4 g	0.1 g
ARjAg	1 g			0.5 g	2 mL	0.4 g	0.1 g
AMAg	1 g	3.5 g	2 mL	0.5 g	2 mL	0.4 g	0.1 g

## Data Availability

The original contributions presented in this study are included in the article. Further inquiries can be directed to the corresponding author.

## Supplementary Materials

The following supporting information can be downloaded at: https://www.mdpi.com/article/10.3390/ijms26146809/s1.

## References

[1] Maeso L., Antezana P.E., Arana A.G.H., Evelson P.A., Orive G., Desimone M.F. (2024). Progress in the Use of Hydrogels for Antioxidant Delivery in Skin Wounds. Pharmaceutics.

[2] Iacopetti I., Perazzi A., Martinello T., Gemignani F., Patruno M. (2020). Hyaluronic acid, Manuka honey and Acemannan gel: Wound-specific applications for skin lesions. Res. Vet. Sci..

[3] Oprica G.M., Panaitescu D.M., Lixandru B.E., Usurelu C.D., Gabor A.R., Nicolae C.A., Fierascu R.C., Frone A.N. (2023). Plant-Derived Nanocellulose with Antibacterial Activity for Wound Healing Dressing. Pharmaceutics.

[4] Lai J.N., Azad A., Sulaiman W.M.A.W., Kumarasamy V., Subramaniyan V., Alshehade S.A. (2024). Alginate-Based Encapsulation Fabrication Technique for Drug Delivery: An Updated Review of Particle Type, Formulation Technique, Pharmaceutical Ingredient, and Targeted Delivery System. Pharmaceutics.

[5] Zhang Q., Huang Z., Jiang H., Wu M., Dong Z., Chen C., Chen F., Zhao G., Ma P. (2025). “Bamboo-like” strong and tough sodium alginate/polyacrylate hydrogel fiber with directional controlled release for wound healing promotion. Carbohydr. Polym..

[6] Duceac I.A., Verestiuc L., Dimitriu C.D., Maier V., Coseri S. (2020). Design and Preparation of New Multifunctional Hydrogels Based on Chitosan/Acrylic Polymers for Drug Delivery and Wound Dressing Applications. Polymers.

[7] Albuquerque P.B.S., de Oliveira W.F., Dos Santos Silva P.M., Dos Santos Correia M.T., Kennedy J.F., Coelho L. (2022). Skincare application of medicinal plant polysaccharides—A review. Carbohydr. Polym..

[8] Stavarache C., Garea S.A., Ghebaur A., Iovu H. (2023). K-Carrageenan/Sodium Alginate Interpenetrating Network Beads for the Incorporation of Ketoprofen as a Potential Drug Delivery System. UPB Sci. Bull. Ser. B.

[9] Rizi E.M., Ebrahimian-Hosseinabadi M., Kharazi A.Z. (2025). Royal jelly-enriched alginate/gelatin hydrogel film for effective treatment of chronic skin wounds. Mater. Today Commun..

[10] Motelica L., Ficai D., Oprea O.C., Ficai A., Ene V.L., Vasile B.S., Andronescu E., Holban A.M. (2021). Antibacterial Biodegradable Films Based on Alginate with Silver Nanoparticles and Lemongrass Essential Oil-Innovative Packaging for Cheese. Nanomaterials.

[11] Alven S., Aderibigbe B.A. (2020). Chitosan and Cellulose-Based Hydrogels for Wound Management. Int. J. Mol. Sci..

[12] Golmohammadi R., Peerayeh S., Moghadam T., Hosseini S. (2020). Synergistic Antibacterial Activity and Wound Healing Properties of Selenium-Chitosan-Mupirocin Nanohybrid System: An in Vivo Study on Rat Diabetic Staphylococcus aureus Wound Infection Model. Sci. Rep..

[13] Andjic M., Bradic J., Kocovic A., Simic M., Krstonosic V., Capo I., Jakovljevic V., Lazarevic N. (2024). Immortelle Essential-Oil-Enriched Hydrogel for Diabetic Wound Repair: Development, Characterization, and In Vivo Efficacy Assessment. Pharmaceutics.

[14] Rybka M., Mazurek Ł., Konop M. (2022). Beneficial Effect of Wound Dressings Containing Silver and Silver Nanoparticles in Wound Healing-From Experimental Studies to Clinical Practice. Life.

[15] Sabira O., Drisya N., Ajaykumar A.P., Mathew A., Jayaraj K.N., Binitha V.S., Zeena K.V., Roy K.B., Janish P.A., Sheena P. (2024). From Ficus recemosa Leaf Galls to Therapeutic Silver Nanoparticles: Antibacterial and Anticancer Applications. Pharmaceutics.

[16] Nandhini S.N., Sisubalan N., Vijayan A., Karthikeyan C., Gnanaraj M., Gideon D.A.M., Jebastin T., Varaprasad K., Sadiku R. (2023). Recent advances in green synthesized nanoparticles for bactericidal and wound healing applications. Heliyon.

[17] Eid N., Yosri N., El-Seedi H.R., Awad H.M., Emam H.E. (2023). Ag@Sidr honey nanocomposite: Chemical profiles, antioxidant and microbicide procurator. Biocatal. Agric. Biotechnol..

[18] Salvo J., Sandoval C., Schencke C., Acevedo F., del Sol M. (2023). Healing Effect of a Nano-Functionalized Medical-Grade Honey for the Treatment of Infected Wounds. Pharmaceutics.

[19] Ewunkem A., Johnson N., Beard A.l., Tshimanga I., Justice B., Meixner J. (2024). Synthesis of Silver Nanoparticles from Honeybees and Its Antibacterial Potential. Open J. Med. Microbiol..

[20] Orsolic N., Jembrek M.J. (2024). Royal Jelly: Biological Action and Health Benefits. Int. J. Mol. Sci..

[21] Viteri R., Zacconi F., Montenegro G., Giordano A. (2021). Bioactive compounds in *Apis mellifera* monofloral honeys. J. Food Sci..

[22] Martinello M., Mutinelli F. (2021). Antioxidant Activity in Bee Products: A Review. Antioxidants.

[23] Alarjani K.M., Yehia H.M., Badr A.N., Ali H.S., Al-Masoud A.H., Alhaqbani S.M., Alkhatib S.A., Rady A.M. (2023). Anti-MRSA and Biological Activities of Propolis Concentrations Loaded to Chitosan Nanoemulsion for Pharmaceutics Applications. Pharmaceutics.

[24] El-Sakhawy M., Salama A., Tohamy H.S. (2023). Applications of propolis-based materials in wound healing. Arch. Dermatol. Res..

[25] Guo J., Wang Z., Chen Y., Cao J., Tian W., Ma B., Dong Y. (2021). Active components and biological functions of royal jelly. J. Funct. Foods.

[26] Al-Hatamleh M.A.I., Alshaer W., Hatmal M.M., Lambuk L., Ahmed N., Mustafa M.Z., Low S.C., Jaafar J., Ferji K., Six J.L. (2022). Applications of Alginate-Based Nanomaterials in Enhancing the Therapeutic Effects of Bee Products. Front. Mol. Biosci..

[27] Khan K., Malik K., Ahmad M., Qureshi R., Aziz M.A., Gul S., Al-Qahtani W.H., Khan R. (2024). Diversity of melliferous Flora (Apiaries) in Honey and microscopic authentication using LM and SEM Techniques. Flora.

[28] Motelica L., Ficai D., Ficai A., Trusca R.D., Ilie C.I., Oprea O.C., Andronescu E. (2020). Innovative Antimicrobial Chitosan/ZnO/Ag NPs/Citronella Essential Oil Nanocomposite—Potential Coating for Grapes. Foods.

[29] Cárdenas-Escudero J., Galán-Madruga D., Cáceres J.O. (2023). FTIR-ATR detection method for emerging C3-plants-derivated adulterants in honey: Beet, dates, and carob syrups. Talanta.

[30] Lacatusu I., Badea N., Murariu A., Oprea O., Bojin D., Meghea A. (2013). Antioxidant Activity of Solid Lipid Nanoparticles Loaded with Umbelliferone. Soft Mater..

[31] Liu F., Liu Y.C., Guo Y.L., Liu J.R., Dong J.W., Wang T.B., Hao D., Zhang Y.Q. (2024). FTIR determination of the degree of molar substitution for hydroxypropyl chitosan. Carbohyd. Polym..

[32] Hong T., Yin J.Y., Nie S.P., Xie M.Y. (2021). Applications of infrared spectroscopy in polysaccharide structural analysis: Progress, challenge and perspective. Food Chem. X.

[33] Chen D., Guo C., Lu W., Zhang C., Xiao C. (2023). Rapid quantification of royal jelly quality by mid-infrared spectroscopy coupled with backpropagation neural network. Food Chem..

[34] Hashemirad F.-S., Behfar M., Kavoosi G. (2024). Proximate composition, physico-chemical, techno-functional, amino acid profile, fatty acid profile, nutritional quality, antioxidant, anti-amylase and anti-lipase properties of bee bread, royal jelly, and bee propolis. LWT.

[35] Borah R., Ninakanti R., Bals S., Verbruggen S.W. (2022). Plasmon resonance of gold and silver nanoparticle arrays in the Kretschmann (attenuated total reflectance) vs. direct incidence configuration. Sci. Rep..

[36] Wiley B.J., Im S.H., Li Z.Y., McLellan J., Siekkinen A., Xia Y.N. (2006). Maneuvering the surface plasmon resonance of silver nanostructures through shape-controlled synthesis. J. Phys. Chem. B.

[37] Alzoubi F.Y., Ahmad A.A., Aljarrah I.A., Migdadi A.B., Al-Bataineh Q.M. (2023). Localize surface plasmon resonance of silver nanoparticles using Mie theory. J. Mater. Sci. Mater. Electron..

[38] Amirjani A., Firouzi F., Haghshenas D.F. (2020). Predicting the Size of Silver Nanoparticles from Their Optical Properties. Plasmonics.

[39] Motelica L., Ficai D., Petrisor G., Oprea O.C., Trusca R.D., Ficai A., Andronescu E., Hudita A., Holban A.M. (2024). Antimicrobial Hydroxyethyl-Cellulose-Based Composite Films with Zinc Oxide and Mesoporous Silica Loaded with Cinnamon Essential Oil. Pharmaceutics.

[40] Motelica L., Ficai D., Oprea O., Ficai A., Trusca R.D., Andronescu E., Holban A.M. (2021). Biodegradable Alginate Films with ZnO Nanoparticles and Citronella Essential Oil—A Novel Antimicrobial Structure. Pharmaceutics.

[41] Luo J.D., Xie Z.L., Lam J.W.Y., Cheng L., Chen H.Y., Qiu C.F., Kwok H.S., Zhan X.W., Liu Y.Q., Zhu D.B. (2001). Aggregation-induced emission of 1-methyl-1,2,3,4,5-pentaphenylsilole. Chem. Commun..

[42] Zhang H.K., Tang B. (2021). Through-Space Interactions in Clusteroluminescence. JACS Au.

[43] Yuan W.Z., Zhang Y.M. (2017). Nonconventional Macromolecular Luminogens with Aggregation-Induced Emission Characteristics. J. Polym. Sci. Polym. Chem..

[44] Gong Y.Y., Tan Y.Q., Mei J., Zhang Y.R., Yuan W.Z., Zhang Y.M., Sun J.Z., Tang B.Z. (2013). Room temperature phosphorescence from natural products: Crystallization matters. Sci. China Chem..

[45] Geng Z.G., Zhang H.M., Xiong Q.Z., Zhang Y.X., Zhao H.J., Wang G.Z. (2015). A fluorescent chitosan hydrogel detection platform for the sensitive and selective determination of trace mercury(II) in water. J. Mater. Chem. A.

[46] Dou X.Y., Zhou Q., Chen X.H., Tan Y.Q., He X., Lu P., Sui K.Y., Tang B.Z., Zhang Y.M., Yuan W.Z. (2018). Clustering-Triggered Emission and Persistent Room Temperature Phosphorescence of Sodium Alginate. Biomacromolecules.

[47] Yu J.Z., Hu N., Hou L.R., Hang F.X., Li K., Xie C.F. (2023). Effect of deacetylation of chitosan on the physicochemical, antioxidant and antibacterial properties activities of chitosan-mannose derivatives. J. Sci. Food Agric..

[48] Bardajee G.R., Mahmoodian H., Amiri B., Atashkadi M. (2025). Immobilization of CdTe QDs on Glucose-Imprinted Alg-g-P(AA-co-VPBA) Nanocomposite for Optical Sensing of Glucose. Langmuir.

[49] Dumitru C.D., Neacsu I.A., Oprea O.C., Motelica L., Voicu Balasea B., Ilie C.-I., Marinescu F., Ripszky A., Pituru S.-M., Andronescu E. (2025). Biomaterials Based on Bee Products and Their Effectiveness in Soft Tissue Regeneration. Materials.

[50] Iwuji C., Saha H., Ghann W., Dotson D., Bhuiya M.A.K., Parvez M.S., Jahangir Z.M.G.S., Rahman M.M., Chowdhury F.I., Uddin J. (2024). Synthesis and characterization of silver nanoparticles and their promising antimicrobial effects. Chem. Phys. Impact.

[51] Aldakheel F., Wickramasinghe R., Thamaraiselvan C., Sayed M., Fagir M., Dein D., Mohsen D. (2025). Green silver nanoparticle-embedded chitosan-alginate hydrogel: A novel antibacterial approach for potential wound healing. Polym. Polym. Compos..

[52] Oe T., Dechojarassri D., Kakinoki S., Kawasaki H., Furuike T., Tamura H. (2023). Microwave-Assisted Incorporation of AgNP into Chitosan-Alginate Hydrogels for Antimicrobial Applications. J. Funct. Biomater..

[53] Obeidat M., Haddad M.A., Ghnamat S.A. (2024). Antimicrobial activities of seasonally collected bee products: Honey, propolis, royal jelly, venom, and mellitin. Braz. J. Biol..

[54] Yanovska A., Husak Y., Korniienko V., Viktoriia H., Mishchenko O., Banasiuk R., Radwan-Pragłowska J., Piatkowski M., Janus Ł., Maksym P. (2021). Development, characterization and antimicrobial properties of silver nanoparticles loaded chitosan-alginate sponges for biomedical application. J. Mater. Res..

[55] Vanti G., Poondla N., Manogaran P., Teradal N., S V., Kaulgud R., Kurjogi M. (2024). Synthesis and Characterization of Multifunctional Chitosan–Silver Nanoparticles: An In-Vitro Approach for Biomedical Applications. Pharmaceuticals.

[56] Nalbantsoy A., Varol E., Çaglar A., Yücel B. (2024). Cytotoxic and apoptotic effectiveness of Cypriot honeybee (*Apis mellifera cypria*) venom on various cancer cells. Turk. J. Biochem..

[57] Bonamigo T., Campos J.F., Alfredo T.M., Balestieri J.B., Cardoso C.A., Paredes-Gamero E.J., de Picoli Souza K., Dos Santos E.L. (2017). Antioxidant, Cytotoxic, and Toxic Activities of Propolis from Two Native Bees in Brazil: *Scaptotrigona depilis* and *Melipona quadrifasciata* anthidioides. Oxidative Med. Cell. Longev..

[58] Sharma J.N., Al-Omran A., Parvathy S.S. (2007). Role of nitric oxide in inflammatory diseases. Inflammopharmacology.

[59] Pahlavani N., Malekahmadi M., Firouzi S., Rostami D., Alireza S., Bagheri Moghadam A., Ferns G., Navashenaq J., Rezvani R., Safarian M. (2020). Molecular and cellular mechanisms of the effects of Propolis in inflammation, oxidative stress and glycemic control in chronic diseases. Nutr. Metab..

[60] Kurek-Górecka A., Górecki M., Rzepecka-Stojko A., Balwierz R., Stojko J. (2020). Bee Products in Dermatology and Skin Care. Molecules.

[61] Zuo W., Wei K., Zhang X., Wang D., Gong H., Zhang Y., Wang H. (2024). A Multifunctional Nanozyme Hydrogel with Antibacterial, Antioxidative, and Photo-Induced Nitric Oxide-Supplying Properties for Promoting Infected Wound Healing. Pharmaceutics.

[62] Man M.Q., Wakefield J.S., Mauro T.M., Elias P.M. (2022). Regulatory Role of Nitric Oxide in Cutaneous Inflammation. Inflammation.

[63] Liang H., He X., Li X., Semiruomi D., Yan F. (2023). Effect of Royal Gel addition to chitosan matrix for wound dress applications: Fabrication, characterization and artificial neural network analysis. Environ. Technol. Innov..

[64] Choudhary P., Tushir S., Bala M., Sharma S., Sangha M., Rani H., Yewle N., Kumar P., Singla D., Chandran D. (2023). Exploring the Potential of Bee-Derived Antioxidants for Maintaining Oral Hygiene and Dental Health: A Comprehensive Review. Antioxidants.

[65] Lesmana R., Zulhendri F., Fearnley J., Irsyam I.A., Rasyid R.P.H.N., Abidin T., Abdulah R., Suwantika A., Paradkar A., Budiman A.S. (2022). The Suitability of Propolis as a Bioactive Component of Biomaterials. Front. Pharmacol..

[66] Zara S., Fioravanti G., Ciuffreda A., Annicchiarico C., Quaresima R., Mastrangelo F. (2023). Evaluation of Human Gingival Fibroblasts (HGFs) Behavior on Innovative Laser Colored Titanium Surfaces. Materials.

[67] Ghasemi M., Turnbull T., Sebastian S., Kempson I. (2021). The MTT Assay: Utility, Limitations, Pitfalls, and Interpretation in Bulk and Single-Cell Analysis. Int. J. Mol. Sci..

[68] Froelich A., Jakubowska E., Wojtyłko M., Jadach B., Gackowski M., Gadziński P., Napierała O., Ravliv Y., Osmałek T. (2023). Alginate-Based Materials Loaded with Nanoparticles in Wound Healing. Pharmaceutics.

[69] (2021). Performance Standards for Antimicrobial Susceptibility Testing.

[70] Motelica L., Vasile B.-S., Ficai A., Surdu V.-A., Ficai D., Oprea O.C., Andronescu E., Mustățea G., Ungureanu E.L., Dobre A.A. (2023). Antibacterial activity of zinc oxide nanoparticles loaded with essential oils. Pharmaceutics.

