# Supplementary material for: Antimicrobial Composite Films Based on Alginate–Chitosan with Honey, Propolis, Royal Jelly and Green-Synthesized Silver Nanoparticles
Source: Int J Mol Sci. 2025 Jul 16;26(14):6809. doi: 10.3390/ijms26146809 (PMC12295747; doi:10.3390/ijms26146809)
Supplement: Supplementary file 1 [file ijms-26-06809-s001.zip › ijms-3731089-supplementary.pdf]

International Journal of
Molecular Sciences

MDPI

Article

Antimicrobial composite films based on alginate - chitosan with honey, propolis, royal jelly and green-synthesized silver nanoparticles

Corina Dana Dumitru, Cornelia-Ioana Ilie, Ionela Andreea Neacsu, Ludmila Motelica, Ovidiu Cristian Oprea, Alexandra Ripszky, Silviu Mirel Pițuru, Bianca Voicu Bălașea, Florica Marinescu, Ecaterina Andronescu

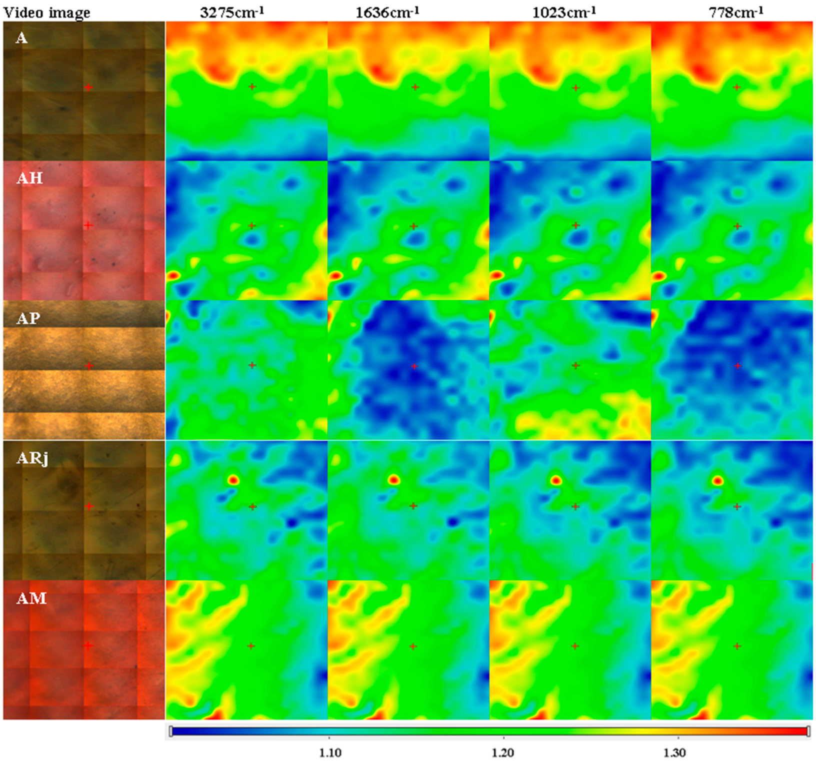

Figure S1. FTIR maps for the samples without AgNPs (A; AH; AP; ARj and AM)

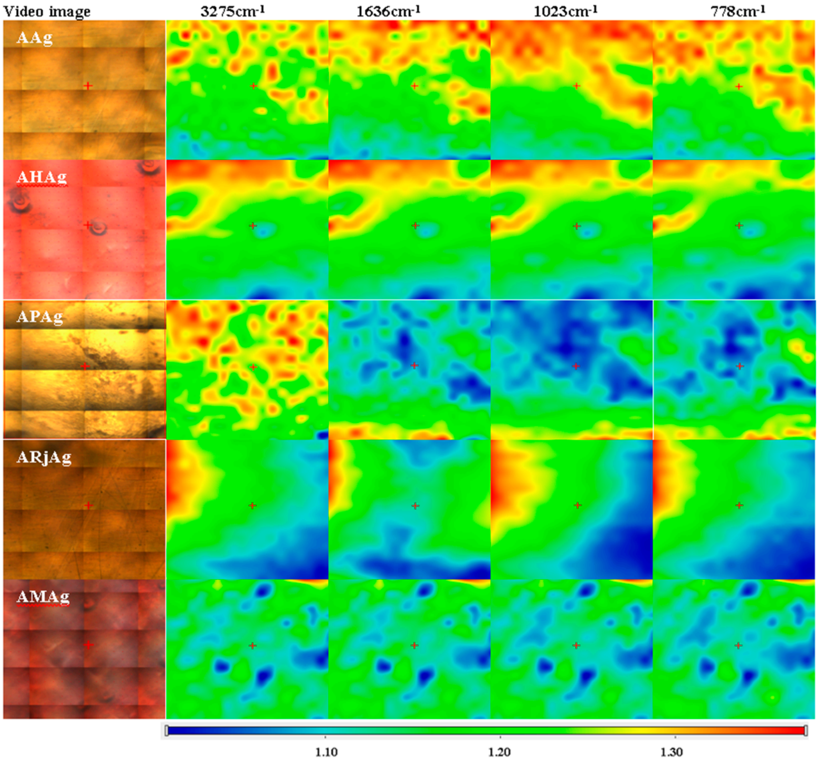

Figure S2. FTIR maps for the samples with AgNPs (AAg; AHAg; APAg; ARjAg and AMAg)

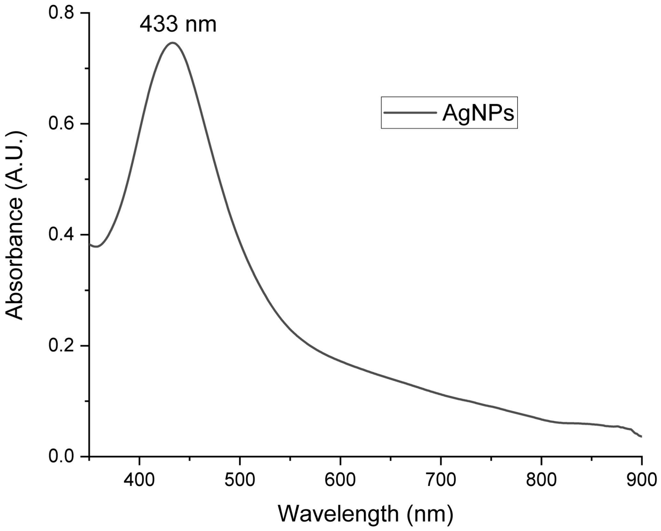

Figure S3. UV-Vis spectra for the AgNPs colloidal suspension

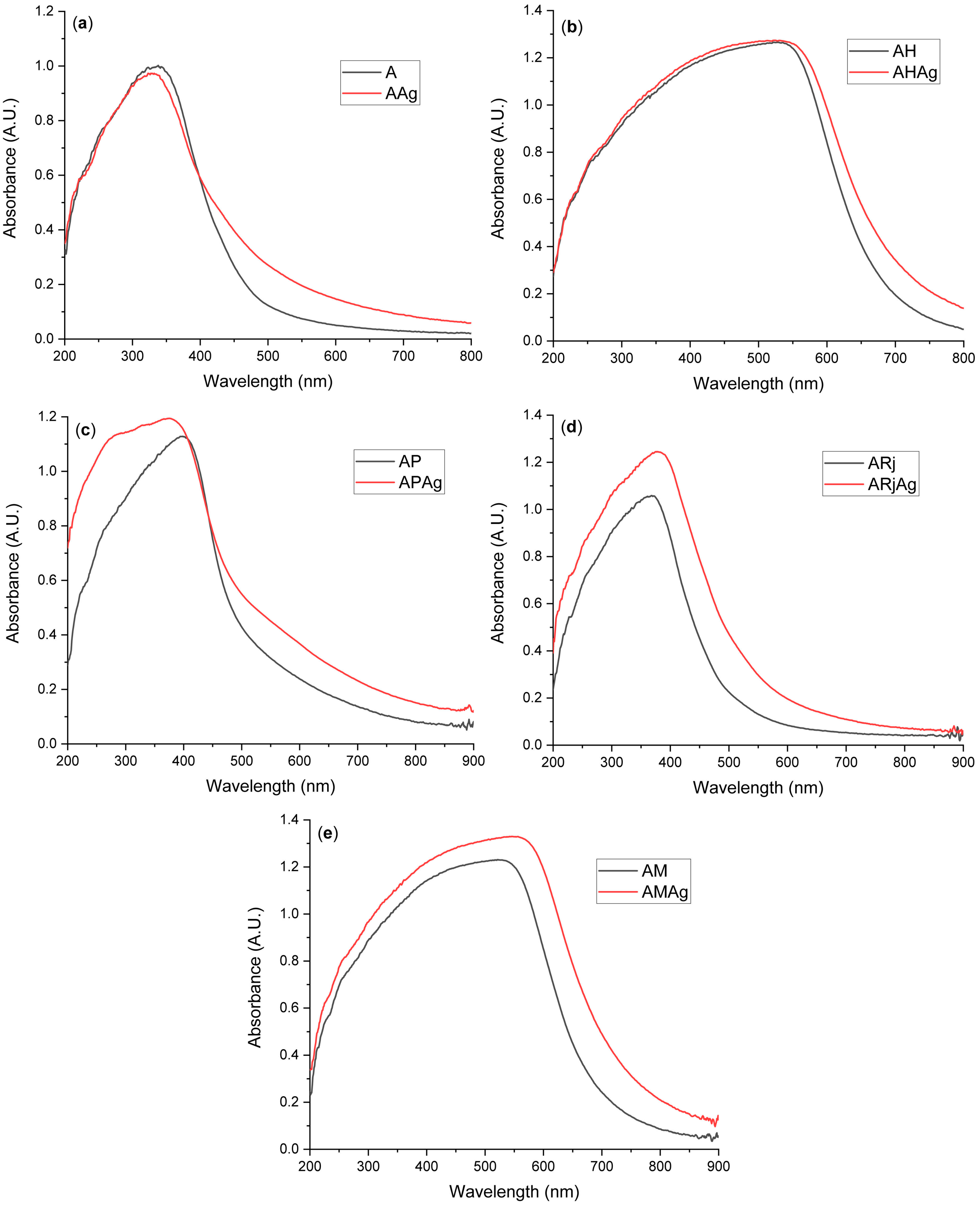

Figure S4. Comparison between UV-Vis spectra for the samples with AgNPs (red lines) and without AgNPs (black lines): A and AAg (**a**); AH and AHAg (**b**); AP and APAg (**c**); ARj and ARjAg (**d**); AM and AMAg (**e**).

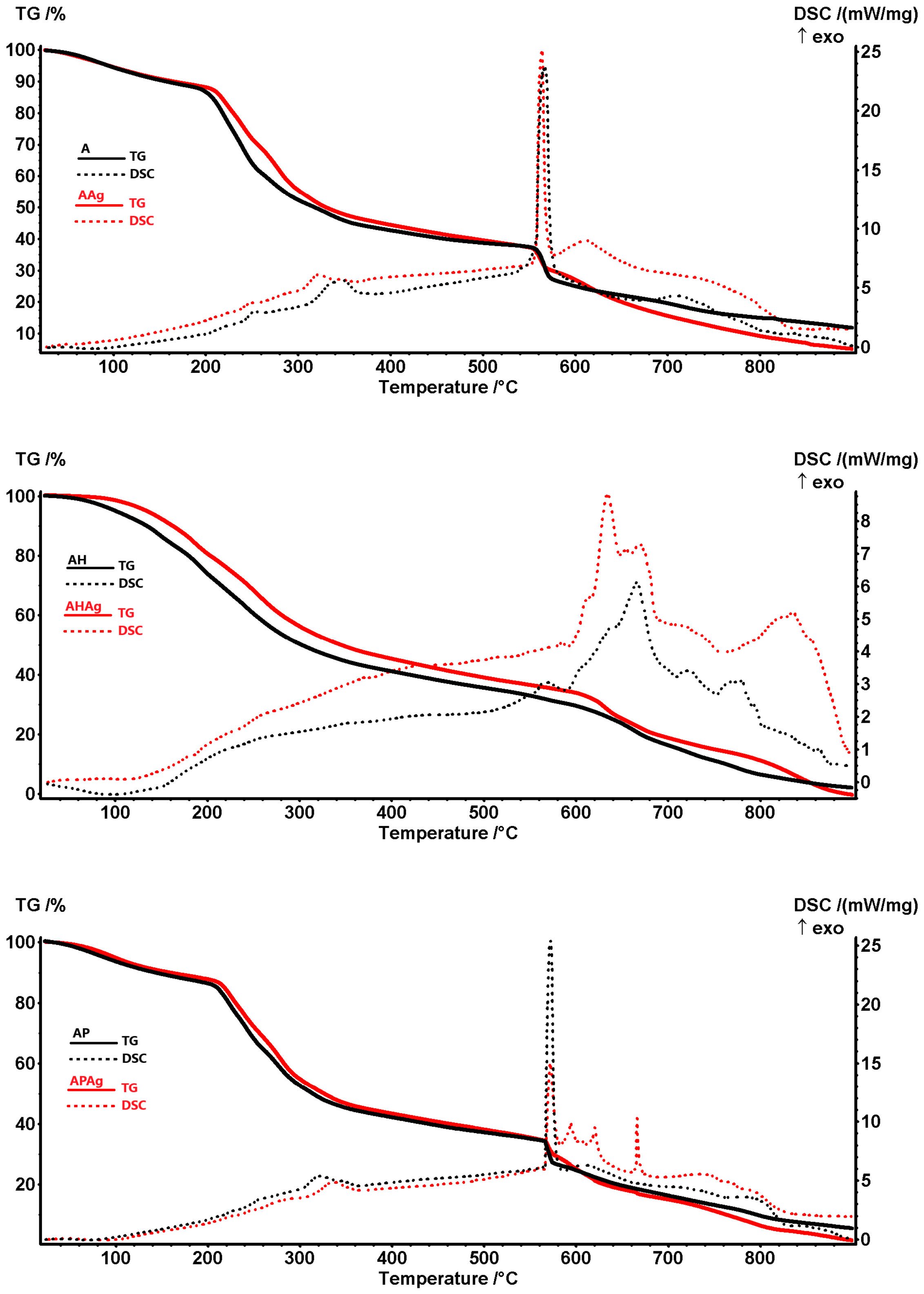
TG /%
DSC /(mW/mg)
↑ exo
A TG
DSC
AAg TG
DSC
Temperature /°C
TG /%
DSC /(mW/mg)
↑ exo
AH TG
DSC
AHAg TG
DSC
Temperature /°C
TG /%
DSC /(mW/mg)
↑ exo
AP TG
DSC
APAg TG
DSC
Temperature /°C

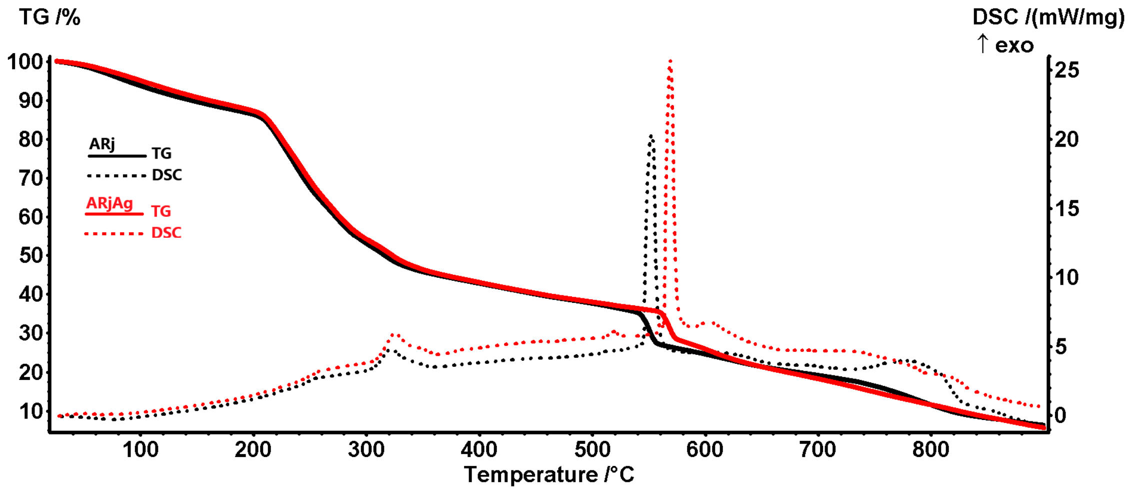

Figure S5. Comparative thermal analyses (TG and DSC curves) for samples without (black curves) and with AgNPs (red curves). The TG curves are represented as solid lines, while DSC curves are represented as dotted lines
